# A GIS workflow for the identification of corridors of geomorphic river recovery across landscapes

**DOI:** 10.1371/journal.pone.0278831

**Published:** 2022-12-13

**Authors:** Danelle Agnew, Bradley P. Graves, Kirstie Fryirs

**Affiliations:** School of Natural Sciences, Macquarie University, North Ryde, NSW, Australia; University of Bucharest, ROMANIA

## Abstract

The provision of a simplified GIS workflow to analyse the Open Access NSW River Styles database provides non-technical GIS users in river management with the ability to quickly and efficiently obtain information to assist them in catchment-scale rehabilitation prioritisation. Publicly available proprietary GIS software, standard GIS tools, and a packaged digital elevation model are used to demonstrate the ease of analysis for those with some GIS skills, to establish where corridors of geomorphic river recovery occur or could be built at-scale. Rather than a ‘single use’ report, this novel application of GIS methods is designed to be used by those responsible for river management, replicated across landscapes and adjusted according to preferences. Decision making becomes more cost effective, and adaptive to local circumstances and changing river management priorities. The method could also be adjusted and applied to other river monitoring and condition datasets where polyline data layers are available.

## Introduction

Geographic Information Systems (GIS) analysis has become an important tool for analysing riverine environments at different scales and with significant resolution [1–4]. However, it is often the case that expert knowledge and spatial analysis expertise is required to work with and manipulate GIS datasets. This can severely limit the accessibility and use of available data to those with the GIS skills to undertake such analyses, creating a significant barrier to information and use of best-available science in practice [5–8]. Similarly, the increasing complexity of GIS environments and tools, and the speed at which they evolve, can hinder the usability of spatial analysis if the tools and processes are not easily understood by the end-user [9, 10]. As such, it is critical that the workflows that sit behind these analyses are made publicly available, not only for interrogation and peer-review but so end-users of results can consider the foundation principles and decisions that go into the analysis, thereby providing a basis for understanding the usefulness of the outputs produced.

The power of GIS lies in its ability to enable a greater understanding of the natural environment, by maximising and enhancing the use of existing datasets [11]. For example, environmental management agencies and organisations often hold vast volumes of monitoring and evaluation data (e.g., vegetation mapping, ecological data) in a GIS environment that could be better used for a range of different purposes, including improving decision-support and prioritisation approaches [12, 13]. This is particularly relevant in the multi-billion dollar global industry of river management and restoration where resource allocation decisions are made daily. Having evidence-based systems to support decision making and prioritisation in a consistent and transparent manner is critical to the integrity of the river management industry. This is particularly important when resources–either financial or human–are limited and need to be allocated wisely.

There is a growing number of semi-automated workflows for the geomorphic analysis of river systems [14–19]. These can provide workflows based on proprietary GIS software toolkits (ArcGIS and TopoToolbox) and python script, which are ideally suited to expert GIS users. For non-technical GIS users, many of these workflows and analyses are impenetrable. The provision of simplified workflows using standard tools in readily available proprietary or open source GIS software, such as ArcGIS [20] or GRASS GIS [21], respectively, is critical if the industry is to make best use of available datasets.

Agnew and Fryirs [22] uses the NSW River Styles database to identify and quantify where corridors of river recovery may be established as part of process- and nature-based approaches to river management. The NSW River Styles database contains extensive river characterisation information for over 216,000 km of stream length across the State of New South Wales (NSW) Australia, including a geomorphic recovery potential layer [23, 24]. Geomorphic recovery potential is defined in the River Styles Framework as the likelihood there being an improvement in geomorphic river condition over a 50–100 year management period [25–27]. Recovery potential occurs along a continuum of classes starting with Conservation and Strategic, and followed by declining recovery potential from High Recovery Potential (HRP), to Moderate Recovery Potential (MRP) and Low Recovery Potential (LRP) [22, 28]. The findings presented in Agnew and Fryirs [22] highlight the potential of using the state-wide NSW River Styles database in a new and novel way, in practice, to prioritise where corridors of river recovery can be created in catchments. The novelty of the GIS workflow developed is its analysis of patterns and sequences in polyline data. This is done by examining relationships between locations and attributes of data identified by geographic position on Earth [29]. It analyses and connects polyline shapefiles according to their recovery potential class. While the recovery potential layer of the NSW River Styles database has been used in this workflow, the same principles and workflow can be applied to any database that contains polyline shapefiles.

In this paper we provide a GIS workflow method:

1. to identify corridors of river recovery from the River Styles database.
2. that non-technical GIS users can use to identify reaches in a catchment for rehabilitation prioritisation.
3. that goes beyond a ‘single use’ analysis, and can be replicated across catchments and sub-catchments as needed.
4. that the user has the ability to apply or adjust based on user-defined parameters.
5. which is applicable to other large-scale monitoring and evaluation datasets.

The GIS workflow has been used to identify corridors of river recovery for all NSW coastal catchments as reported in Agnew and Fryirs [22]. In Agnew and Fryirs [22], 13 different combinations of reach and loci connections are identified using the protocol. The step-by-step GIS workflow is publicly available as an open access protocol with protocols.io.

**Fig 1:**
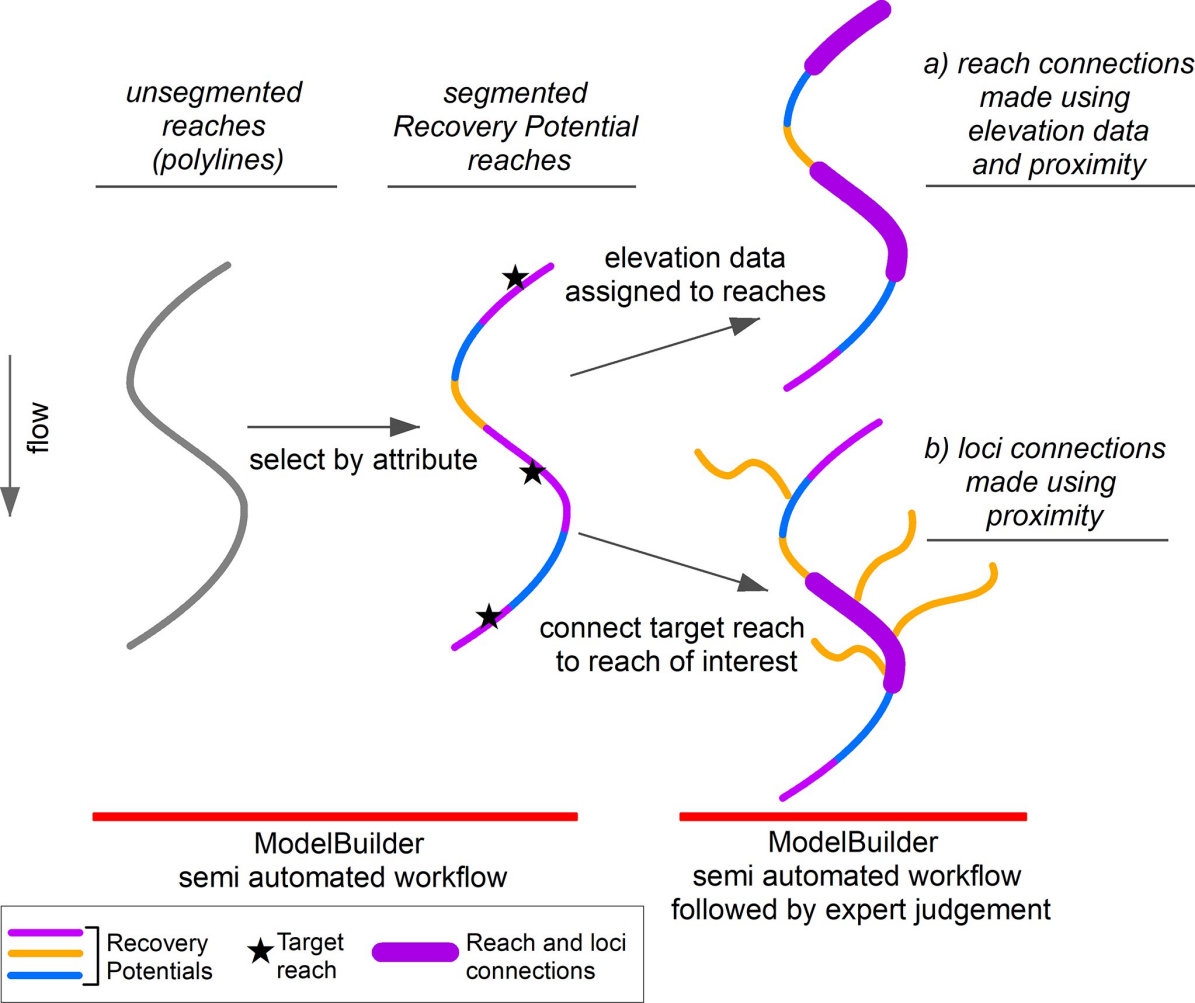
Schematic representation of River Styles reaches (polylines) to create reach and loci connections. In a) reach connections are shown in the thicker purple line upstream of blue reaches (the reaches of interest) and in b) loci connections are shown in the thicker purple line, surrounded by orange reaches (the reaches of interest). Data source: River Styles Database [23], ArcMap [20], and 30 m SRTM DEM-H [30].

The workflow has been developed using existing GIS software and tools with the explicit purpose to aid or assist a non-technical GIS user. The workflow is quick, efficient and streamlined. For this analysis, the geomorphic recovery potential layer in the NSW River Styles database [23], and a publicly available packaged digital elevation model (DEM), are used and analysed using ArcMap release 10.8 [20]. River reaches in the NSW River Styles database are delineated as polylines. Analysis of polyline connections based on proximity is a standard procedure within ArcMap. Here, we analyse polylines based on their recovery potential class and their proximity to each other to identify ‘reach’ and ‘loci’ connections that create corridors of river recovery. In Agnew and Fryirs [22] reach connections are defined as an upstream to downstream section of a river that is connected end-to-end. Loci are isolated river sections, where more complex connections link trunk streams with their surrounding tributaries. Recovery can be seeded from these reaches into adjacent reaches. In Agnew and Fryirs [22] loci connections are defined as upstream to downstream sections of a river that are connected both end-to-end within an individual stream, and directly linked to tributaries. Fig 1 shows a schematic representation of reach and loci connections.

## Materials and methods

The protocol described in this peer-reviewed article is published on protocols.io, https://dx.doi.org/10.17504/protocols.io.n2bvj8625gk5/v1 and is included for printing as Supporting Information file named S1 File with this article.

## Expected results

### The corridor workflow

**Fig 2:**
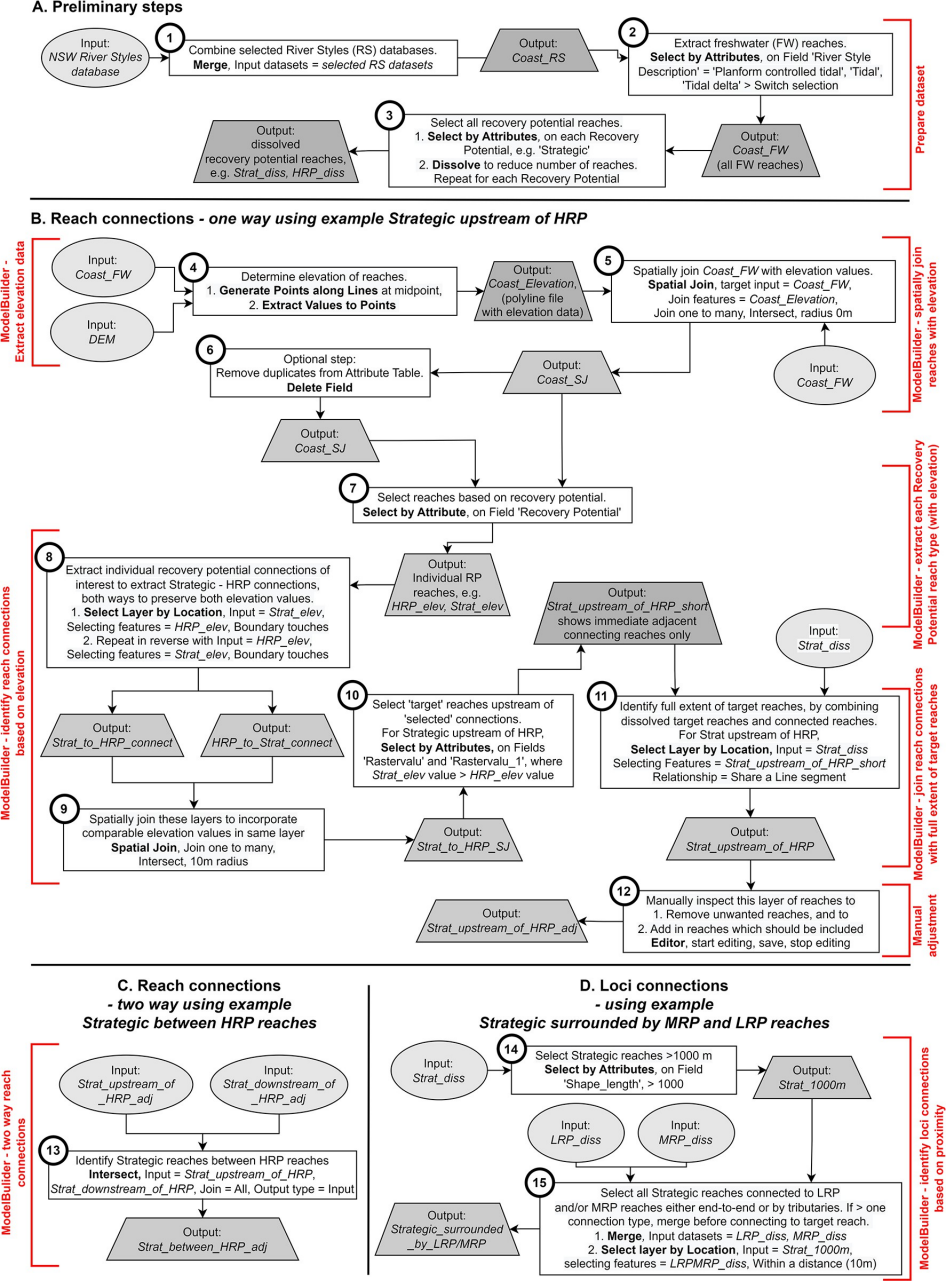
Workflow to identify reach and loci connections. Extraction of a one-way reach connection (Strategic reach upstream of HRP reach), and loci connection (Strategic reach surrounded by LRP and MRP reaches). Data source: River Styles Database [23], ArcMap [20], and 30 m SRTM DEM-H [30].

The full workflow for the identification of reach and loci connections to build corridors of river recovery is shown in Fig 2. The workflow uses ModelBuilder in ArcMap 10.8.

### Data pre-processing before using the corridor workflow

GIS analysis was performed in ArcMap version 10.8 [20], using the NSW River Styles database and a packaged DEM [22]. The River Styles database contains freshwater and tidal reaches across NSW, and is organised into regions, with some regions comprising several catchments. For the case study in Agnew and Fryirs [22], the regions containing freshwater and tidal reaches for NSW’s coastal catchments were extracted from the state-wide database, covering an area of 129,222 km^2^. The coastal regions comprise almost 120,000 freshwater reaches, ranging in length from <0.001 km to ~105 km, averaging 0.7 km, and totalling almost 85,000km of stream length.

To decrease GIS processing time, reaches with identical recovery potential occurring adjacent to each other along single stream lines were merged, creating almost 42,000 reaches ranging between <0.001 km up to ~155 km in length.

The NSW River Styles database does not contain any elevation data associated with the polyline shapefiles. An Open Access DEM was used to provide elevation data for the analysis. Elevation data is used to determine which reaches are upstream and flow into downstream reaches that sit at lower elevation. From this, the pattern and sequencing of reaches can be processed and mapped. The target recovery potential reaches and connections of interest can be displayed (Fig 1).

Choice of DEM is an important consideration for two reasons; it should be appropriate for the scale of analysis being undertaken (i.e., consistent and complete coverage for catchments under investigation), and also the complexity of processes applied [31]. Firstly, given that corridor analysis is normally conducted at a regional or catchment scale, and along tens of thousands of kilometres of stream length, a high resolution DEM is not required. The use of high resolution DEMs, for example, 1 m resolution Light Detection and Ranging (LiDAR) derived DEMs, require longer and computationally intensive processing time, considerable manual manipulation and mosaicking of individual tiles to provide a region or catchment wide DEM, and are more suited to those practitioners with specialised GIS skills. Here, the single download, 30 m resolution pre-processed and packaged DEM, provides an appropriate tool for cost effective, timely, meaningful analysis at catchment and regional scales. Secondly, the processes applied use existing GIS software tools (e.g., Spatial Analyst Toolbox), without significant data manipulation or use of programming languages (e.g. python).

To satisfy these two considerations, the publicly available 30 m Shuttle Radar Topography Mission (SRTM) DEM was selected for this study [30]. To determine reach elevation, a single midpoint elevation raster value (m) for each individual polyline was extracted from the DEM. With an average reach length of 0.7 km this resulted in adjoining streamline elevation raster values being 0.7 km apart, maximising the likelihood of a downstream decrease in elevation being detected between adjacent reaches using the 30 m resolution DEM. The DEM’s elevation data was then visually examined and compared to the NSW River Styles network of streams to check that the upstream-downstream sequences of reaches was correct [14].

### Running the corridor workflow

To run the workflow to identify reach and loci connections, the process involves identifying a ‘target’ reach, which is selected based on its recovery potential class and suitability for rehabilitation (Fig 1) [22]. Reach and loci connections are then determined based on their proximity (i.e. where the polylines abut) to this ‘target’ reach (Fig 1). For loci connections, a minimum threshold reach length of 1 km was used to reduce the number of connections made.

Reach connections require identification of end-to-end connections, along a single streamline comprised of several recovery potential classified polylines. The loci connections require identification of upstream-downstream and end-to-end connections, as well as tributary connections to the trunk stream. In the database, individual reach polyline features are determined by attributes including River Style, stream condition and recovery potential. Reaches which are adjacent to each other may have an identical recovery potential, but other attributes which are different, and are therefore categorised as separate features. For corridor analysis and prioritisation purposes, it is important to understand the full longitudinal extent of a target recovery potential reach along a streamline. Therefore, reaches along a streamline with identical recovery potential need to be joined.

**Fig 3:**
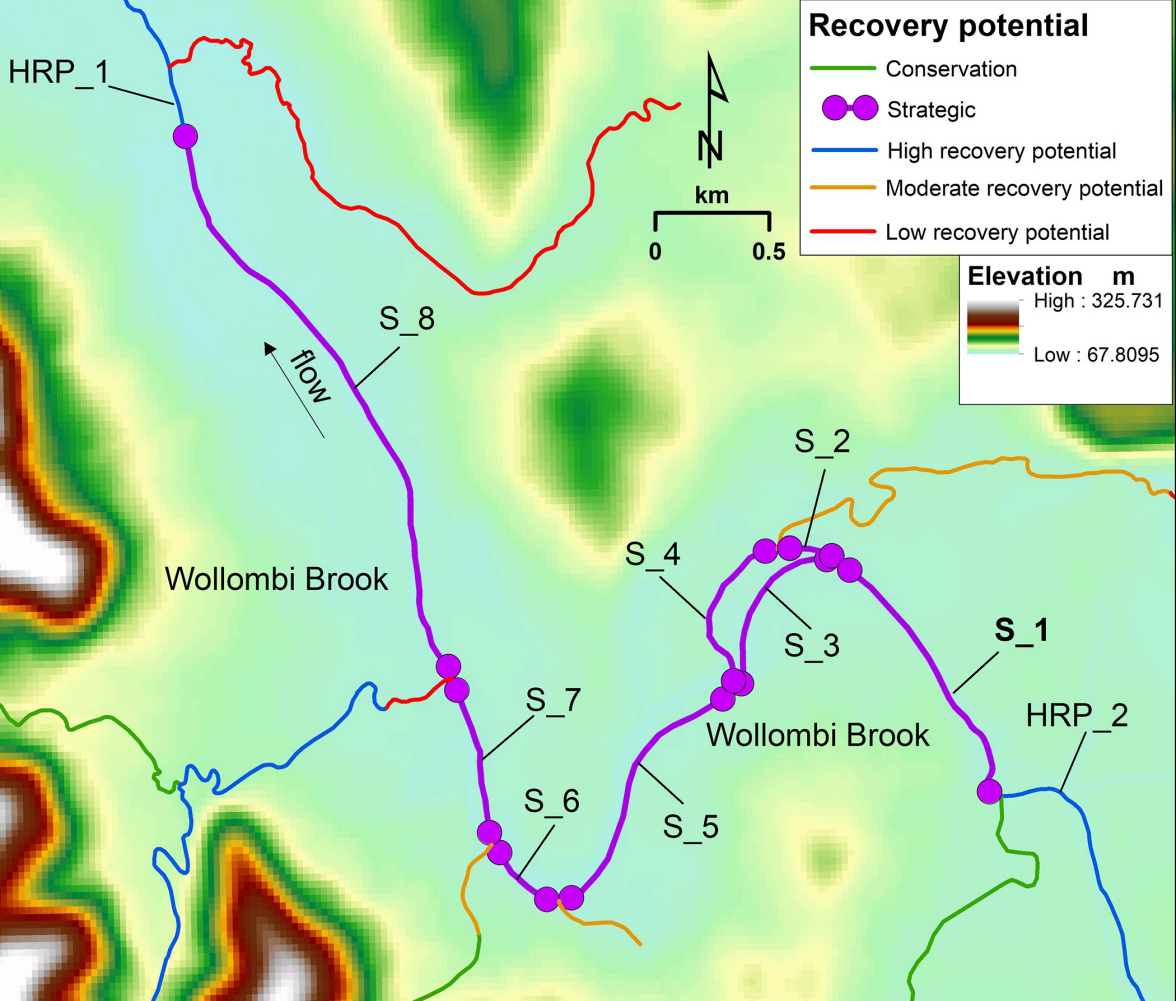
Strategic recovery potential section of Wollombi Brook. The image shows eight single Strategic features (S_1 to S_8) connected end-to-end which form one long Strategic reach. Data source: River Styles Database [23], ArcMap [20], and 30 m SRTM DEM-H [30].

To identify a reach connection, the linkage position between a targeted recovery potential feature (e.g. Strategic) and the connection of interest (e.g. High Recovery Potential (HRP)) needs to be located in the database. This connection only occurs at the point where two different recovery potential polyline features join at one end of their respective polylines. This creates a polyline connection between a single targeted recovery potential feature and the connection of interest (i.e. using the example, Strategic connected to HRP). This does not consider the full extent of the target recovery potential reach (e.g. Strategic) that is adjacent in both upstream and downstream directions. Once a connection (for example, Strategic upstream of HRP) is made using two different recovery potential classes, then this connection must be extended to the identical adjoining targeted recovery potential features (e.g. Strategic) to measure the full extent of the recovery potential connection which has been made. An example is shown in Fig 3 for Wollombi Brook (a catchment in the Hunter Valley) where eight Strategic polyline features (S_1 to S_8) occur end-to-end, with a HRP reach (HRP_1) immediately downstream of S_8. The workflow will make a connection between HRP_1 and S_8 immediately upstream. These will be connected to the remaining S_1 to S_7 in the workflow to form an extended Strategic reach (S_1 to S_8) upstream of HRP_1.

This same workflow can be employed to identify alternative connections that may be of interest to a user by using different ‘target’ recovery potential reaches connected to other recovery potential classes of interest. It can be used to extract reaches of interest which are situated between selected reaches, for example, Strategic reaches between HRP reaches.

### Expected outputs and verification

**Fig 4:**
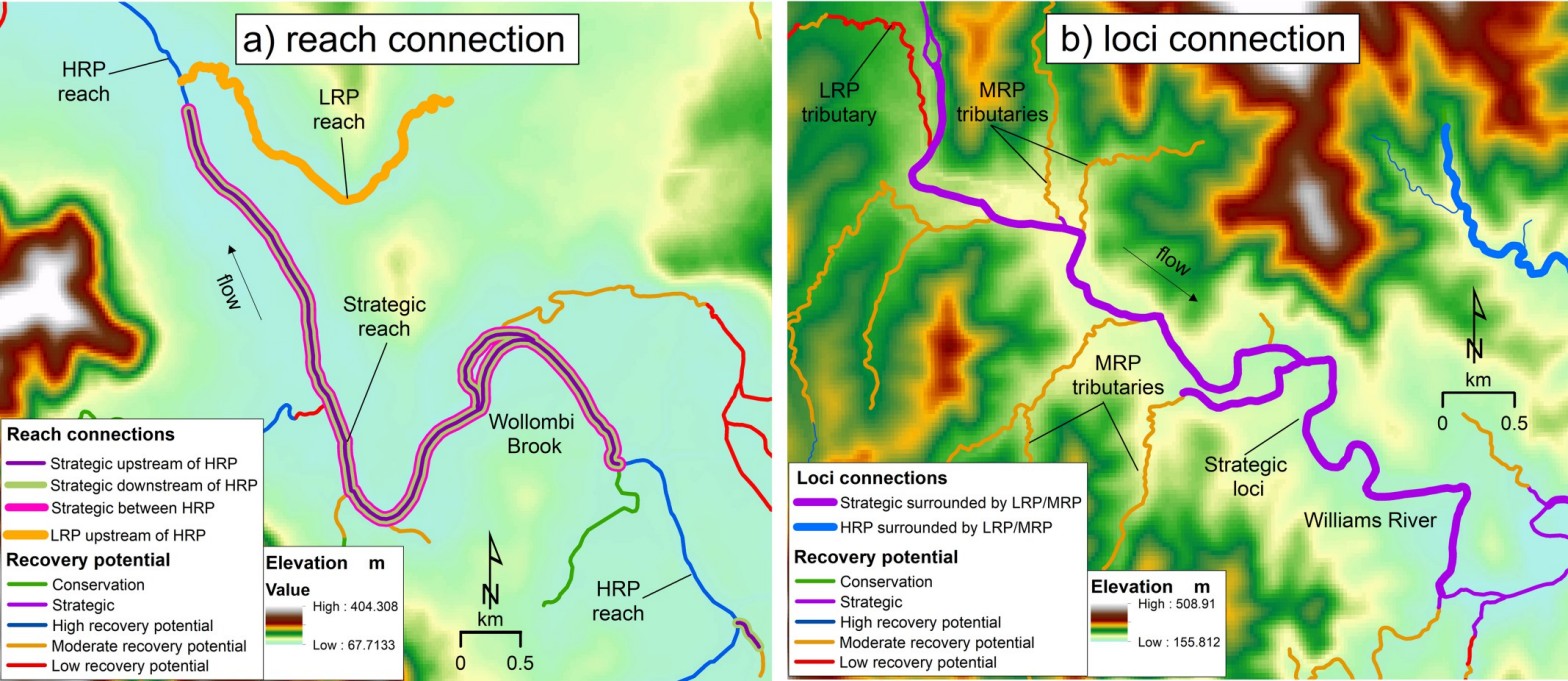
Examples of reach and loci connections Shown are a) two reach connections, showing a target Strategic reach between two HRP reaches, and a target LRP reach upstream of an HRP reach, on Wollombi Brook in the Hunter catchment, and b) a loci connection, showing a target Strategic reach on the Williams River, in the Hunter catchment, surrounded by LRP and MRP reaches.

Output from the workflow can identify selected reach and loci connections. Here for display purposes, Wollombi Brook and the Williams River in the Hunter catchment are used to demonstrate the workflow output (Fig 4).

For the Wollombi Brook example, a user is interested in identifying two different reach connections. They want to firstly identify where Strategic recovery potential reaches occur between two HRP reaches, and secondly, identify where LRP reaches occur upstream of HRP reaches. Therefore reaches with either a Strategic recovery potential or LRP recovery potential classification become the target reaches, and HRP recovery potential reaches become the selected connections of interest. Running the workflow identifies a Strategic between HRP reach connection along the trunk stream and an LRP reach in a tributary upstream of a HRP reach located on the trunk stream (Fig 4A).

For the Williams River example, a user is interested in identifying loci connections. They want to identify where Strategic recovery potential reaches are surrounded by MRP and LRP reaches. In this case, reaches with a Strategic recovery potential classification become the target reaches, and reaches with MRP and LRP become the selected connections of interest. Running the workflow identifies a Strategic loci surrounded by LRP and/or MRP along the trunk stream that could be used to focus rehabilitation (Fig 4B).

In our study, to verify the output produced from the workflow, all reach and loci connections identified were manually inspected using the NSW River Styles database. ModelBuilder identified ~70% of reach and loci connections. To improve performance, several adjustments and checks should be made. First, check that the positions of streamlines in the database are positioned on the valley bottom in the DEM. For example, mapped streams may track along the DEM hillslope rather than the valley bottom, producing incorrect data when extracting elevation from the DEMs. However, at the scale of the average stream length of 0.7 km relative to the DEM resolution, the method generally returned correct data. For short reaches, where there was misalignment of the River Styles polyline with the DEM valley bottom, manual adjustment was required. Second, where there were gaps between reach polylines, these needed to be connected. Third, elevation values were determined from the DEM based on the midpoint of each reach. This resulted in the midpoint elevation value of longer target reaches being higher relative to a reach confluence or tributary connection occurring at a lower elevation in a downstream location along the target reach. To fix this, consideration was given to dissolving reaches with identical recovery potential and using these longer reaches to derive elevation data. However, this resulted in long reaches where the midpoint elevation produced incorrect results when connecting with confluences or tributaries towards the upstream end of the merged reaches. Therefore, it was more appropriate to use single feature classes to provide more accurate elevation data relative to confluences and tributaries entering the main trunk stream along the reach. Finally, short ‘null’ reaches occurred where the recovery potential of the river had not been assessed. Short null reaches occurring adjacent to target reaches prevented potentially appropriate reach connections (assessed by visual inspection) being made via ModelBuilder.

### Conclusion: Need for the corridor workflow

Open access river system datasets such as NSW River Styles are comprehensive repositories of information and critical for river rehabilitation decision support and prioritisation [22, 24]. However, these databases can be overwhelming, containing extensive and detailed analyses of river structure, function and health [24]. The industry needs to make much better use of such resources, but in an efficient and user-friendly way [10]. Here we provide a methodology to enable non-technical GIS users directly involved in river management, to interrogate datasets such as the NSW River Styles database (and others like it in various parts of the world) to produce outputs and results that can inform consistent and transparent decision making and prioritisation for resource allocation [5, 31, 32].

This GIS workflow is a novel approach, in that it significantly simplifies the analytical process. The workflow can run on any polyline data, so is amenable to many spatial datasets, enabling a variety of connections to be made between polylines according to the strategic priorities of river managers. The workflow could also be applied to other river datasets, such as the dataset for the Columbia Habitat Monitoring Program (CHaMP) [33]. The workflow can be run for sub-catchments, catchments, regions or States thereby allowing for cross-scalar applications.

In this Lab Protocol, we have combined the comprehensive NSW River Styles database and a coarse resolution DEM, then undertaken to develop a GIS workflow that easily identifies reaches that can be linked to each other to create corridors of river recovery [22]. The analysis derives results with sufficient accuracy to provide a manageable short list of potential rehabilitation sites for prioritisation [24, 31]. It uses inbuilt tools within readily available proprietary GIS software such as ArcMap, without the need for programming language such as python. Importantly, rather than a ‘single use’ analysis, our workflow can be replicated across catchments, sub-catchments and regions with the user choosing the ‘target’ reach recovery potential class to run.

Whilst some manual adjustment may be required at the end of the process, this forms part of the necessary vetting process of the short list of reaches and loci connections identified by the workflow. Decision making is thus more adaptive to local circumstances and evolving river management priorities, using the latest information available, in a more cost effective manner.

The results from use of the workflow that are reported in Agnew and Fryirs [22] demonstrate its effectiveness in identifying a prioritised shortlist of reach and loci connections across all coastal catchments of NSW. Approximately 4,900 km and 17,400 km of targeted reach and loci connections, respectively, have been distilled from almost 85,000 km of coastal freshwater stream length.

The corridor identification workflow presented in this paper can be used to systematically rank reaches for rehabilitation based on geomorphic recovery potential. The analysis can be extended to determine a range of recovery potential connections and patterns within catchments, and rank them according to broader priorities of river managers. River managers can consider other connections that may be a priority for management. This technique of linking sections of river together into corridors enables consideration of rehabilitation options based on position, pattern and sequence [34]. Importantly, these distilled shortlists can be combined with river managers’ local catchment knowledge and strategic priorities, grounding the decision making in reality. Creation of this layer of reach and loci connections can be integrated with other prioritisation considerations, for example, societal, economic and other catchment priorities as an initial step towards achieving more holistic and sustainable rehabilitation outcomes at the landscape scale [11, 35].

## Supporting information

S1 File This file available on protocols.io. (PDF)
