# Supplementary material for: A GIS workflow for the identification of corridors of geomorphic river recovery across landscapes
Source: PLoS One. 2022 Dec 13;17(12):e0278831. doi: 10.1371/journal.pone.0278831 (PMC9746969; doi:10.1371/journal.pone.0278831)
Supplement: S1 File — (PDF) [file pone.0278831.s001.pdf]

A GIS workflow for the identification of corridors of geomorphic river recovery across landscapes

COMMENTS 0

RESERVED DOI:
10.17504/protocols.io.n2bvj8625gk5/v1

Danelle Agnew[1], Bradley Graves[1],
Kirstie Fryirs[1]

[1]Macquarie University

Danelle Agnew

WORKS FOR ME 1

ABSTRACT

The provision of a simplified GIS workflow to analyse the Open Access NSW River Styles database provides non-technical GIS users in river management with the ability to quickly and efficiently obtain information to assist them in catchment-scale rehabilitation prioritisation. Publicly available GIS software, and a digital elevation model (DEM) were used to demonstrate the ease of analysis for those with some GIS skills, to establish where corridors of geomorphic river recovery occur or could be built at-scale. Rather than a 'single use' report, this novel application of GIS methods is designed to be used by those responsible for river management, replicated across landscapes and adjusted according to preferences. Decision making becomes more cost effective, and adaptive to local circumstances and changing river management priorities. The method could also be adjusted and applied to other river monitoring and condition databases.

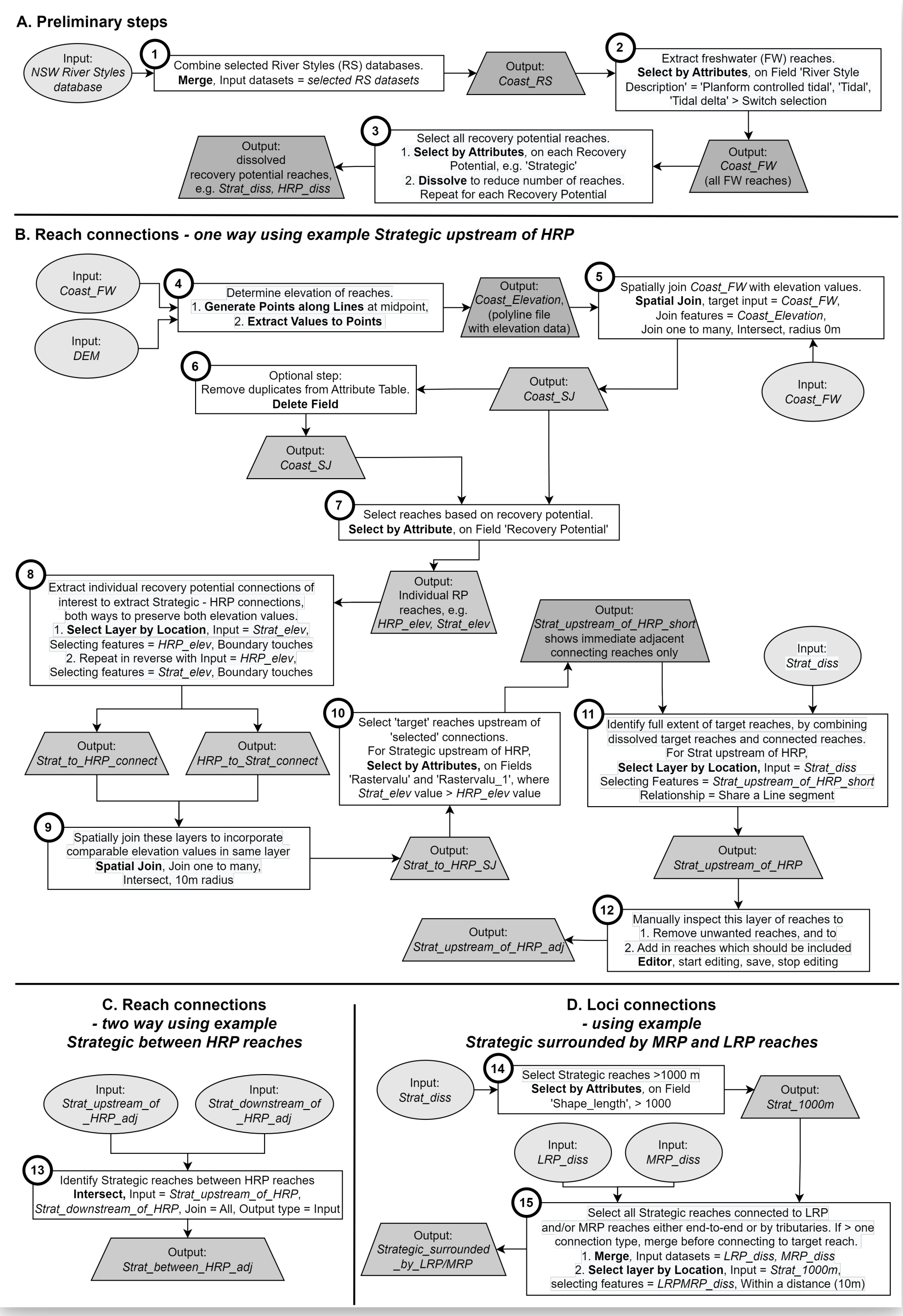

Flowchart to identify reach and loci connections

PROTOCOL INFO

Danelle Agnew, Bradley Graves, Kirstie Fryirs . A GIS workflow for the identification of corridors of geomorphic river recovery across landscapes. **protocols.io**
https://protocols.io/view/a-gis-workflow-for-the-identification-of-corridors-ch9ft93n

KEYWORDS

GIS workflow, river rehabilitation, recovery potential, corridor analysis

CREATED

Oct 21, 2022

LAST MODIFIED

Oct 22, 2022

PROTOCOL INTEGER ID

71687

GUIDELINES

This protocol has been developed using ArcMap for Desktop: Release 10.8. Nevertheless, previous ArcMap versions and other GIS software can be used (e.g. QGIS, GRASS GIS®). If previous versions or different GIS software are being used, some functionalities may have to be accessed and applied differently.

In this protocol, the steps are described using ArcMap, but not within ModelBuilder, as the steps describe examples of the process, rather than the steps for all connection options. If appropriate, the steps in this protocol can be set up and replicated for other selected connections in ModelBuilder.

The accompanying flowchart provides a summary visual representation of the protocol steps.

In the step-by-step protocol, typographical emphases have been used for software as follows:

1. Software processing tools are written in bold, e.g. **Select by Location**
2. Output feature layers are in brackets and written in italics, e.g. *(Coast_FW)*
3. Computational workflows are linked by angle markers (>), e.g. **Spatial Join** > Target feature *(Coast_FW)* > Join Features *(Coast_Elevation)* > Join one to many > Match option = Intersect > Search radius = 0m > Output *(Coast_SJ)*
4. Categories of ArcMap processing tools are in square brackets, e.g. [Spatial Analyst]

Output feature layer names e.g. *(Strat_upstream_of_HRP)*, or *(Strat_elev)* are at the discretion of the user

Abbreviations used in this protocol

Strat Strategic
Con Conservation
HRP High recovery potential
MRP Moderate recovery potential
LRP Low recovery potential
RS River Styles
FW Freshwater

MATERIALS TEXT

1. ArcMap (or similar GIS) software. This protocol uses ArcMap for Desktop release 10.8. ArcGIS software is available from: https://www.esri.com/en-us/home

2. NSW Open Access River Styles database. Available from: https://www.arcgis.com/apps/webappviewer/index.html?id=425c7364e9dc4a71a90c4ba353b8949f
An ArcGIS online account is required to access the database.

3. DEM. This protocol uses 1 second SRTM Derived Hydrological Digital Elevation Model (DEM-H) version 1.0. Record 1.0.4. Available from: https://ecat.ga.gov.au/geonetwork/srv/eng/catalog.search#/metadata/71498

BEFORE STARTING

This workflow uses the examples of extraction of a:

1. one-way reach connection 'Strategic reach upstream of HRP reach',
2. two-way reach connection 'Strategic reach between HRP reaches', and
2. loci connection 'Strategic reach surrounded by MRP and LRP reaches'.

Load the River Styles database and a packaged DEM into ArcMap. The River Styles database is divided into 18 datasets representing 18 regions across NSW. For this workflow, 7 datasets representing NSW coastal regions are used.

A. Preliminary Steps

1 Combine selected River Styles (RS) datasets.

Merge [Data Management] > Input dataset *(selected coastal River Styles polyline feature classes)* > Output dataset *(Coast_RS).*

2 Extract freshwater reaches. Using *(Coast_RS)*, select all freshwater reaches, by removing all tidal reaches, and create a new feature layer.

Open *(Coast_RS)* Attribute Table > in Table options menu, select **Select by Attributes** > Method = Create a new selection > select Attribute field = River Style Description > select Attribute records = 'Planform controlled tidal', 'Tidal' and 'Tidal delta' > Apply > Close > right-click *(Coast_RS)* layer in Table of Contents > Selection > Switch selection > Create layer from selected features > Output layer *(Coast_FW).*

Note

After each processing step, on the Tools toolbar, click Clear Selected Features before commencing the next step.

3 Select all reaches based on recovery potential from *(Coast_FW)*, producing feature layers of each recovery potential. Dissolve each feature layer to link together reaches of the same recovery potential which meet end-to-end.

3.1 Open *(Coast_FW)* Attribute Table > in Table options menu, select **Select by Attributes** > Method = Create a new selection > select Attribute field = Recovery Potential > select Attribute record = 'Strategic' > Apply > Close > right-click *(Coast_FW)* layer in Table of Contents > Selection > Create layer from selected features > Output layer *(Strat_FW)*.

3.2 **Dissolve** [Data Management] > Input features *(Strat_FW)* > uncheck all Dissolve Fields > check Create multipart features and Unsplit lines > Output layer *(Strat_diss)*. Clear selected features before the next step.

Using Attribute record = 'HRP', repeat Steps 3.1 and 3.2 to produce *(HRP_diss)*.

Note

To apply this workflow to other types of connections, repeat Step 3 for Conservation, MRP and LRP Attribute records to produce *(Con_diss)*, *(MRP_diss)*, and *(LRP_diss).*

B. Reach connections - one way

4 Determine the elevation of reaches, by extracting elevation values halfway along each polyline, using the DEM and the coastal freshwater layer *(Coast_FW)*, producing a point feature layer of the midpoint elevation value for each polyline record.

4.1 **Generate Points Along Lines** [Data Management] > Input feature *(Coast_FW)* > Point placement = percentage = 50% > uncheck Include End Points > Output point layer *(Point_Coast)*.

4.2 **Extract values to points** [Spatial Analyst] > Input point features *(Point_Coast)* > Input raster *(DEM)* > Output point layer *(Coast_Elevation)*.

5 Spatially join the coastal freshwater layer with the elevation layer. The spatially joined layer will include an elevation value (i.e. Field = Rastervalu) for each polyline record.

Spatial Join [Analysis] > Target features *(Coast_FW)* > Join Features *(Coast_Elevation)* > Join operation = Join one to many > Match option = Intersect > Search radius = 0m > Output layer *(Coast_SJ)*.

6 Optional Step: Clean up Attribute Table to remove duplicated Fields.

Delete Field [Data Management] > Input Table *(Coast_SJ)* > Drop Field, select required duplicated fields to be removed > Output layer *(Coast_SJ)*.

7

Note

Steps 7-12 are based on the example of Strategic reaches upstream of HRP reaches. Strategic reaches are the 'target' reaches, and HRP reaches are the 'selected connections'.

Select reaches based on recovery potential which also contain elevation values. Extract reaches of each recovery potential from *(Coast_SJ)*, producing output feature layers of *(Strat_elev)* and *(HRP_elev)*.

Open *(Coast_SJ)* Attribute Table > in Table options menu, select **Select by Attributes** > Method = Create a new selection > select Attribute field = Recovery Potential > select Attribute record = 'Strategic' > Apply > Close > right-click *(Coast_SJ)* layer in Table of Contents > Selection > Create layer from selected features > Output layer *(Strat_elev)*. Clear selected features before the next step.

Repeat Step 7 for HRP Attribute records to produce *(HRP_elev)*.

Note

To apply this workflow to other types of connections, repeat Step 7 for Conservation, MRP and LRP Recovery Potential Attribute record to produce *(Con_elev)*, *(MRP_elev)*, and *(LRP_elev)*.

8 Extract individual recovery potential connections of interest (i.e. 'target' reach with 'selected connection'), both ways, to preserve elevation values for both.

8.1 **Select Layer by Location** [Data Management] > Input feature layer *(Strat_elev)* > Relationship = Boundary touches > Selecting features *(HRP_elev)* > Selection Type = New selection > OK > right-click *(Strat_elev)* layer in Table of Contents > Selection > Create layer from selected features > Output *(Strat_to_HRP_connect)*. Clear selected features before the next step.

8.2 Repeat step 8.1, reversing *(HRP_elev)* and *(Strat_elev)*.

Select Layer by Location [Data Management] > Input feature layer *(HRP_elev)* > Relationship = Boundary touches > Selecting features *(Strat_elev)* > Selection Type = New selection > OK > right-click *(HRP_elev)* layer in Table of Contents > Selection > Create layer from selected features > Output *(HRP_to_Strat_connect)*. Clear selected features before the next step.

9 Spatially join these two layers of connections of interest to incorporate comparable elevation values within each Attribute record into the one layer.

Spatial Join [Analysis] > Target feature *(Strat_to_HRP_connect)* > Join Features *(HRP_to_Strat_connect)* > Join operation = Join one to many > Match option = Intersect > Search radius = 10m > Output layer *(Strat_to_HRP_SJ)*.

10 Using elevation values, select the 'target' reaches upstream of the 'selected connections'. Using the 'Strategic upstream of HRP' example, the connection point where an upstream Strategic polyline joins with a downstream HRP polyline is identified. This is where two single polylines join, and does not include the full extent of the target Strategic reaches connected end-to-end in the upstream direction.

Note

In the *(Strat_to_HRP_SJ)* Attribute table, Field 'Rastervalu' refers to the elevation of the 'target' Strategic reach, and 'Rastervalu_1' refers to the elevation of the 'selected connection' HRP reach.

Open *(Strat_to_HRP_SJ)* Attribute Table > in Table options menu, select **Select by Attributes** > Method = Create a new selection > select Attribute fields to create formula 'Rastervalu' '>' 'Rastervalu_1', i.e. 'Rastervalu' of the Strategic reach is greater than 'Rastervalu_1' of the HRP reach > Apply > Close > right-click *(Strat_to_HRP_SJ)* layer in Table of Contents > Selection > Create layer from selected features > Output layer *(Strat_upstream_of_HRP_short)*. Clear selected features before the next step.

Note

If instead of 'upstream', the user wishes to determine Strategic reaches 'downstream' of HRP reaches, then the formula in the **Select by Attributes** processing step would be 'Rastervalu' '<' 'Rastervalu_1', i.e. 'Rastervalu' of the Strategic reach is less than 'Rastervalu_1' of the HRP reach. Output layer would be

(Strat_downstream_of_HRP_short).

11 Identify the full extent of target reaches. Select from the dissolved, extended length target reaches, in this example *(Strat_diss)*, which were identified in Step 3.2. From *(Strat_diss)*, select those reaches which share a line segment with the shorter target connection polylines with elevation, in this example *(Strat_upstream_of_HRP_short)* identified in Step 10. Output is a layer of connections of interest, e.g. Strategic reaches upstream of HRP reaches.

Select layer by location [Data Management] > Input feature layer *(Strat_diss)* > Relationship = Share a line segment > Selecting features *(Strat_upstream_of_HRP_short)* > Selection Type = New selection > OK > right-click *(Strat_diss)* layer in Table of Contents > Selection > Create layer from selected features > Output layer *(Strat_upstream_of_HRP)*. Clear selected features before the next step.

12 Manually inspect the layer *(Strat_upstream_of_HRP)* to verify selected connections of interest. If necessary, manually edit, in Step 12.1 to remove unwanted reach connections and, in Step 12.2 to add reach connections that should have been included in Steps 7-11. For example, those reach connections where DEM elevation data does not align with the flow direction, i.e. an upstream reach (e.g. a swamp) with a lower elevation than its connected downstream reach. Output is a layer of final adjusted reach connections of interest.

12.1 **Editor** > Start editing *(Strat_upstream_of_HRP)* > select unwanted polylines and delete > Save > Stop editing.

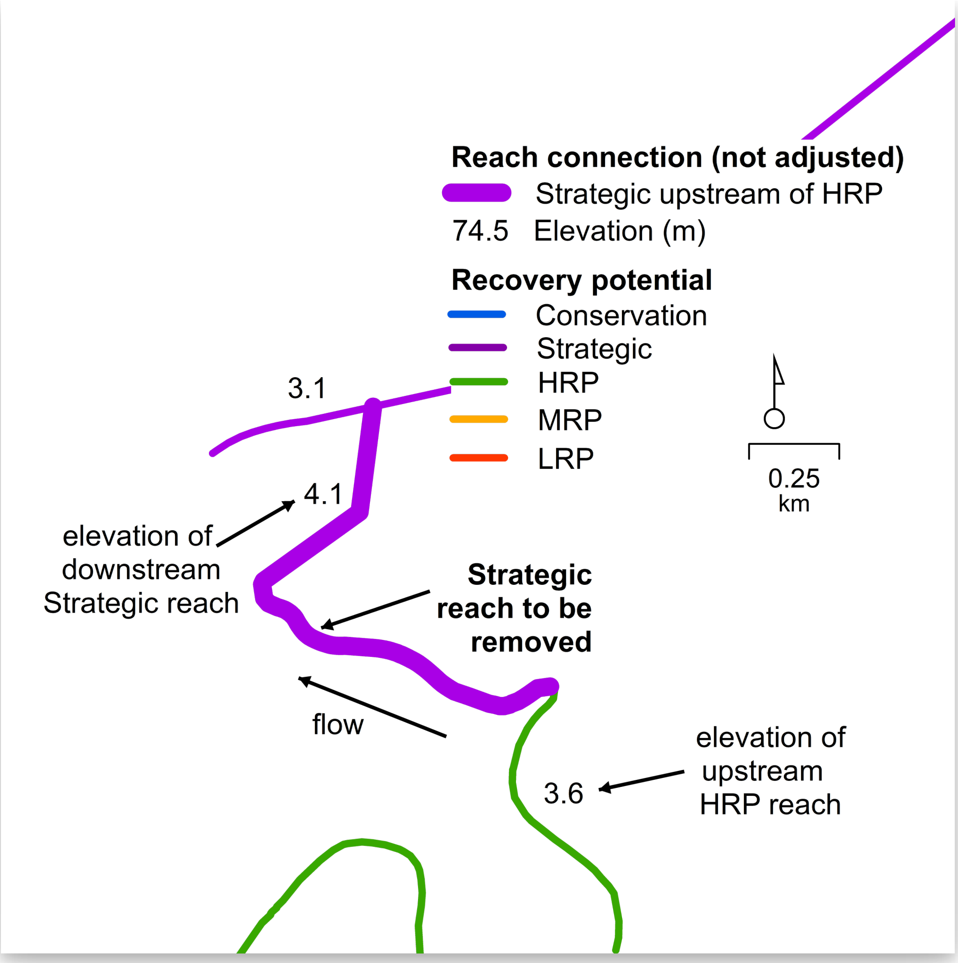

Example of Strategic reach to be removed because its elevation (4.1 m) is higher than the upstream HRP reach elevation (3.6 m).

12.2 In Table of Contents, turn on layers *(Strat_FW)* and *(Strat_upstream_of_HRP)* > Editor > Start editing *(Strat_FW)* > select polylines to add > copy and paste to *(Strat_upstream_of_HRP)* > Save > Stop editing.

Rename layer *(Strat_upstream_of_HRP_adj)*.

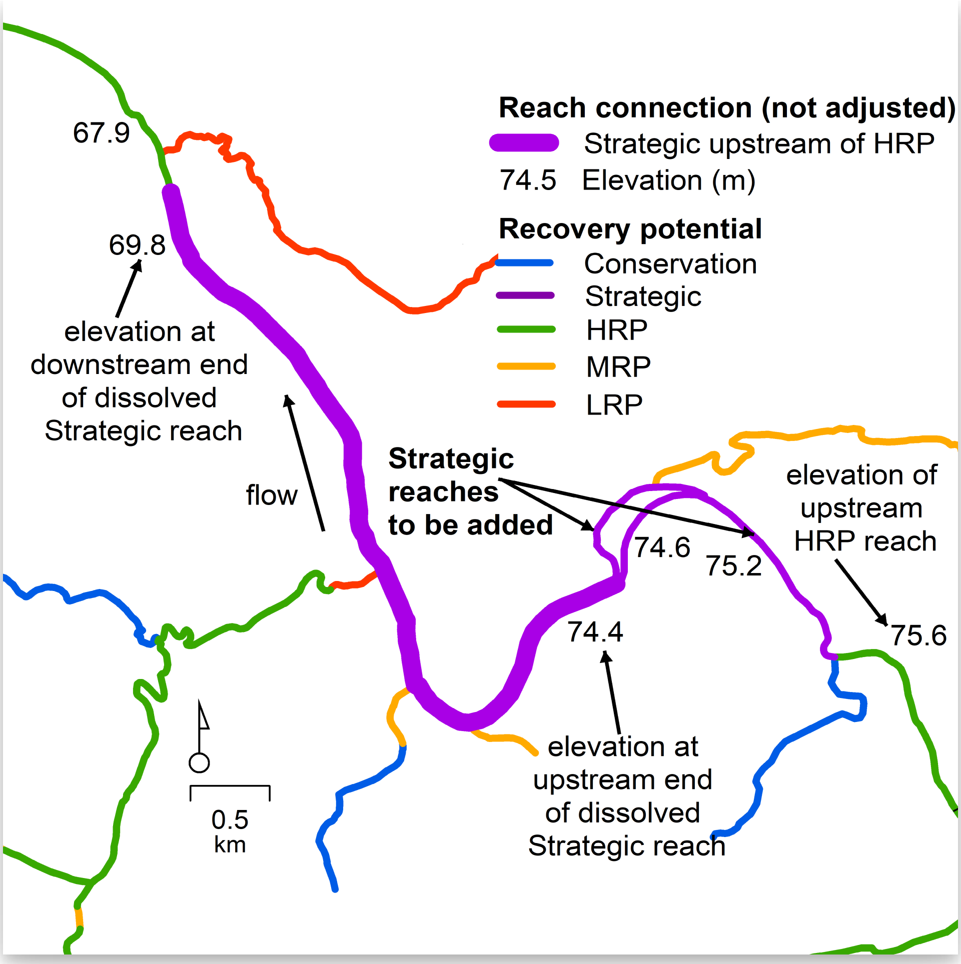

Example of Strategic reaches to be added because their elevations (74.6 and 75.2 m) are lower than the upstream HRP reach elevation (75.6 m).

Expected result

Strategic reaches upstream of HRP reaches (adjusted).

C. Reach connections - two way

13

Note

Step 13 is based on the example of Strategic reaches between HRP reaches, i.e. a target Strategic reach connected to both an upstream HRP reach and a downstream HRP reach. Strategic reaches are the 'target' reaches, and HRP reaches are the 'selected connections'.

To identify where a 'target' reach occurs between two 'selected connections' of interest, identify where the *(Strat_upstream_of_HRP_adj)* layer overlaps with the *(Strat_downstream_of_HRP_adj)* layer. The output is a target reach between two selected connections of interest *(Strat_between_HRP_adj).*

Intersect [Analysis] > Input features *(Strat_upstream_of_HRP_adj)* and *(Strat_downstream_of_HRP_adj)* > Join Attributes = All > Output type = Input > Output layer *(Strat_between_HRP_adj).*

Expected result

Strategic reaches between HRP reaches (adjusted).

D. Loci connections

14

Note

Steps 14-15 are based on the example of Strategic reaches surrounded by MRP and LRP reaches. Strategic reaches are the target reaches, and MRP and LRP reaches are the selected connections.

For loci connections, using the layer of dissolved recovery potential reaches, select 'target' reaches longer than 1000 metres (to ensure loci connections of sufficient length for rehabilitation). Elevation data is not required for loci connections as reach connections both up and downstream and in adjacent tributaries are identified.

Open *(Strat_diss)* Attribute Table > in Table options menu, select **Select by Attributes** > Method = Create a new selection > select Attribute field = 'Shape Length', create formula 'Shape Length' '>' '1000', i.e. Shape Length is greater than 1000 m > Apply > Close > right-click *(Strat_diss)* layer in Table of Contents > Selection > Create layer from selected features > Output layer *(Strat_1000m).*

15 Identify target reaches connected to selected connections, both up and downstream and in adjoining tributaries to the target reach. If there is more than one selected connection type, as in this example, MRP and LRP, then they need to be merged into one layer before connecting to the target reach. Output is the loci connection, i.e. a target reach surrounded by selected connections of interest, which identifies reaches up and downstream, plus tributaries, of the target reach.

15.1 **Merge** [Data Management] > Input datasets *(MRP_diss, LRP_diss)* > Output dataset *(MRPLRP_merge).*

15.2 **Select layer by location** [Data Management] > Input feature layer *(Strat_1000m)* > Relationship = Within a distance > Selecting features *(MRPLRP_merge)* > Search distance = 10m > Selection type = New Selection > OK > Create layer from selected features > Output layer *(Strat_surrounded_by_MRP/LRP).*

Expected result

Strategic reaches surrounded by MRP and LRP reaches
